# Recolonizing gray wolves increase parasite infection risk in their prey

**DOI:** 10.1002/ece3.3839

**Published:** 2018-01-22

**Authors:** Ines Lesniak, Ilja Heckmann, Mathias Franz, Alex D. Greenwood, Emanuel Heitlinger, Heribert Hofer, Oliver Krone

**Affiliations:** ^1^ Leibniz Institute for Zoo and Wildlife Research Berlin Germany; ^2^ Department of Veterinary Medicine Freie Universität Berlin Berlin Germany; ^3^ Ecology and Evolution of Molecular Parasite Host Interactions Humboldt–Universität zu Berlin Berlin Germany; ^4^ Department of Biology, Chemistry, Pharmacy Freie Universität Berlin Berlin Germany

**Keywords:** apicomplexa, coccidia, endoparasites, epidemiology, metabarcoding, protozoa, *Sarcocystis*, ungulates

## Abstract

The recent recolonization of Central Europe by the European gray wolf (*Canis lupus*) provides an opportunity to study the dynamics of parasite transmission for cases when a definitive host returns after a phase of local extinction. We investigated whether a newly established wolf population increased the prevalence of those parasites in ungulate intermediate hosts representing wolf prey, whether some parasite species are particularly well adapted to wolves, and the potential basis for such adaptations. We recorded *Sarcocystis* species richness in wolves and *Sarcocystis* prevalence in ungulates harvested in study sites with and without permanent wolf presence in Germany using microscopy and DNA metabarcoding. *Sarcocystis* prevalence in red deer (*Cervus elaphus*) was significantly higher in wolf areas (79.7%) than in control areas (26.3%) but not in roe deer (*Capreolus capreolus*) (97.2% vs. 90.4%) or wild boar (*Sus scrofa*) (82.8% vs. 64.9%). Of 11 *Sarcocystis* species, *Sarcocystis taeniata* and *Sarcocystis grueneri* occurred more often in wolves than expected from the *Sarcocystis* infection patterns of ungulate prey. Both *Sarcocystis* species showed a higher increase in prevalence in ungulates in wolf areas than other *Sarcocystis* species, suggesting that they are particularly well adapted to wolves, and are examples of “wolf specialists”. *Sarcocystis* species richness in wolves was significantly higher in pups than in adults. “Wolf specialists” persisted during wolf maturation. The results of this study demonstrate that (1) predator–prey interactions influence parasite prevalence, if both predator and prey are part of the parasite life cycle, (2) mesopredators do not necessarily replace the apex predator in parasite transmission dynamics for particular parasites of which the apex predator is the definitive host, even if meso‐ and apex predators were from the same taxonomic family (here: Canidae, e.g., red foxes *Vulpes vulpes*), and (3) age‐dependent immune maturation contributes to the control of protozoan infection in wolves.

## 1 INTRODUCTION

Apex predators play a critical role in shaping food webs (Estes et al., 2011). When a predator is a definitive host of a parasite and disappears from its habitat, it may leave a gap in the food web. Potential consequences of such disappearances for parasite–host relationships are still poorly understood. Even for well‐studied temperate ecosystems, where the gray wolf (*Canis lupus*) is an apex predator and ungulates are its main prey, little is known about the parasitological consequences of a transient wolf removal/extinction (East, Bassano, & Ytrehus, 2011). Transmission dynamics of trophically transmitted pathogens and parasites that are well adapted to a specific host might change. Parasites could either adapt to alternative hosts or disappear overtime (Farrell, Stephens, Berrang‐Ford, Gittleman, & Davies, 2015), which we call the “host flexibility” hypothesis and the “fading out” hypothesis, respectively. Wolves are definitive hosts for a wide range of endoparasites (Craig & Craig, 2005), but little is known about their possible influence on parasite prevalence in their ungulate prey if these serve as intermediate hosts, as in the case of helminths or apicomplexa (Lesniak, Heckmann et al., 2017). In particular, it is unclear whether infection risk increases when a definitive host returns after being absent from a specific area for some time, and how infection risk varies among different prey species. It is also unclear which factors control parasite etiopathology and whether these factors favor specialization of parasites for specific hosts. The process of the current wolf recolonization of Central Europe provides an excellent opportunity to investigate parasite transmission dynamics in a predator–prey system as the same prey species can be examined in the presence and absence of the predator in the same habitat type.

Gray wolves have recolonized parts of Germany and Western Poland since the year 2000 after an absence of nearly 100 years (Reinhardt, Kluth, Nowak, & Myslajek, 2015). In Germany, the first wolf packs settled in the eastern state of Saxony. Since then, the population spread in a northwesterly direction. By 2015, almost 40 packs were recognized in Germany and approximately 70 packs were identified within the entire Central European lowland (CEL) wolf population (Reinhardt et al., 2015). For this population, population structure and dynamics (Ansorge, Holzapfel, Kluth, Reinhardt, & Wagner, 2010; Nowak & Mysłajek, 2016), infectious diseases and causes of death (Szentiks et al., 2016), dispersal (Andersen et al., 2015; Reinhardt et al., 2015), and feeding habits (Nowak, Mysłajek, Kłosińska, & Gabryś, 2011; Wagner, Holzapfel, Kluth, Reinhardt, & Ansorge, 2012) have been investigated since recolonization started. The diet analyses demonstrated that red deer (*Cervus elaphus*), roe deer (*Capreolus capreolus*), and wild boar (*Sus scrofa*) are the three main prey species of the resident wolf population. They may therefore serve as potential intermediate hosts of wolf‐transmitted endoparasites such as helminth metacestodes of *Taenia* spp. (Lesniak, Heckmann et al., 2017) or *Echinococcus* spp. (Onac, Győrke, Oltean, Gavrea, & Cozma, 2013), and protozoan cysts of *Neospora* spp. (Rocchigiani et al., 2016) or *Sarcocystis* spp. (Kolenda, Ugorski, & Bednarski, 2014).

Host–pathogen interactions and epidemiology are well understood in apicomplexan taxa such as *Toxoplasma*—a parasite occurring within a domestic and a sylvatic cycle with zoonotic potential (Shaapan, 2016). However, the links between *Sarcocystis* of wild intermediate and definitive hosts are currently unclear, and the prevalence and distribution of *Sarcocystis* species in ungulates and the potential impact of the removal and then the return of the apex predator are at present unknown.

Free‐ranging wolves from the CEL population host at least 12 different *Sarcocystis* species (Lesniak, Heckmann et al., 2017). The genus *Sarcocystis* has an obligatory two‐host life cycle involving (partially) carnivorous definitive hosts and a broad range of intermediate hosts such as reptiles, birds, or mammals (Dubey & Lindsay, 2006; Munday, Hartley, Harrigan, Presidente, & Obendorf, 1979). *Sarcocystis* are known to be more host‐specific in terms of their intermediate than definitive host range, although the current state of knowledge is far from complete, as new *Sarcocystis* species and new hosts continue to be described (Dahlgren & Gjerde, 2010; Gjerde, 2014b). *Sarcocystis* sexually reproduce in the intestines of their definitive host which sheds sporulated oocysts and sporocysts into the environment during defecation. Grazing intermediate hosts accidentally ingest these infectious stages. The asexual development in the intermediate host begins when *Sarcocystis* penetrate the digestive mucosa and migrate through the blood vessels to reach their target (muscular or nervous) tissue where they eventually form ((sarco–)cysts) (Dubey, Calero_Bernal, Rosenthal, Speer, & Fayer, 2015; Dubey & Lindsay, 2006; Poulsen & Stensvold, 2014). During the early infection phase, pathogenic species may cause clinical symptoms such as weight loss, anemia, fever, and abortion in pregnant intermediate hosts (Buxton, 1998; Dubey & Lindsay, 2006)—otherwise, sarcocystosis usually has an asymptomatic etiopathology and minor impact on its host.

In this study, we investigated the prevalence of *Sarcocystis* in ungulates and wolves. Under the “fading out” hypothesis, returning wolves would reimport temporarily faded parasites, thereby increasing parasite infection risk in ungulate intermediate hosts. It assumes that at least some *Sarcocystis* species are “wolf specialists” and are too host‐specific to use alternative hosts as definitive hosts. If an increase in *Sarcocystis* prevalence occurred, it should therefore be driven by “wolf‐specialized” parasites, that is, *Sarcocystis* species that are particularly well adapted to wolves. “Wolf‐specialized” parasites should then be overrepresented in wolves and show the strongest prevalence increase in ungulate intermediate hosts in wolf‐inhabited areas, and there should be a “mismatch” in relative parasite frequencies between wolves and their prey. Under the “host flexibility” hypothesis, returning wolves serve as an additional definitive host for endemic parasites also spread by other carnivores (spillback) which had resumed the function of alternative hosts (Kelly, Paterson, Townsend, Poulin, & Tompkins, 2009; Moré, Maksimov, Conraths, & Schares, 2016). In this case, we would not expect to find *Sarcocystis* species that could be considered “wolf specialists”. Without “wolf‐specialized” parasites, relative parasite frequencies in ungulate prey species and in wolves would match as wolves would be nonselectively infected with the *Sarcocystis* they consume.

The “fading out” hypothesis also predicts that if parasites are particularly well adapted to a specific host (“wolf specialists”), we would expect them to prevent clearance by the host immune system. Young wolves are likely to be more susceptible to apicomplexan *Sarcocystis* parasites than older animals (in terms of less previous parasite exposure, gradual building up of immunity and hence a generally weaker immune response). Younger wolves would therefore be expected to exhibit a higher *Sarcocystis* species richness than adult wolves. In adults, an improved immune competence should allow them to clear parasites that might infect pups, except for “wolf specialists” which might employ adaptations that allow them to circumvent the host immune system and persist in older individuals. If no age‐related immune processes controlled parasite resistance in wolves, wolves of all ages should host the same *Sarcocystis* community as each pack member is exposed to the same *Sarcocystis* species when they share an infected kill.

## 2 MATERIAL AND METHODS

### 2.1 Sample collection

Ungulate muscle tissue samples (tongue, diaphragm, heart) originating from wolf territories (WT, German federal states of Brandenburg and Saxony, 50°10′–53°33′ N and 11°14′–15°2′ E) or the control area (CA, German federal state of Schleswig–Holstein, 53°20′–54°55′ N and 8°36′–11°7′ E) where no territorial wolves occurred during the sampling period (Figure S1) were collected between November 2012 and December 2014. Red deer (*n*_WT_ = 75, *n*_CA_ = 18), roe deer (*n*_WT_ = 99, *n*_CA_ = 72), and wild boar (*n*_WT_ = 83, *n*_CA_ = 37), shot during hunts and intended for food consumption, were screened. Ungulate age classes (juveniles, subadults, adults) were estimated by hunters.

Forty‐three wolf carcasses collected between 2007 and 2014 were examined for the presence of intestinal *Sarcocystis* spp. Wolves were collected as roadkills or as confiscated poached animals originating from five federal states in northern and eastern Germany (50°10′–54°54′ N and 6°41′–15°2′ E). Wolf age classes (pup, yearling, adult) were determined as previously described (Lesniak, Heckmann et al., 2017).

### 2.2 Ungulate muscle histology

Fresh ungulate muscle tissue samples were fixed in 4% formalin and then embedded in paraffin blocks. Paraffin‐embedded blocks were sectioned at 3 μm, stained with hematoxylin–eosin, and examined by light microscopy to determine *Sarcocystis* sp. presence.

### 2.3 DNA extraction, PCR, and library preparation

DNA from ungulate specimen was isolated using the Invisorb^®^ Spin DNA Extraction Kit (STRATEC Molecular, Berlin, Germany) according to the manufacturer's instructions. DNA eluates (tongue, diaphragm, heart) were pooled per individual for subsequent PCR screening. *Sarcocystis* 18S rRNA gene amplification was performed using a set of three primer pairs (proti15F: 5′–TGCCAGTAGTCATATGCTTGTYT–3′, proti440R: 5′–CAGGCYCSCTCTCCGGA–3′ (Lesniak, Heckmann et al., 2017), SarAF: 5′–CTGGTTGATCCTGCCAGTAG–3′, SarAR: 5′–TTCCCATCATTCCAATCACT–3′, SarBF: 5′–GGGAGGTAGTGACAAGAAATAACAA–3′, SarBR: 5′–GGCAAATGCTTTCGCAGTAG–3′ (both primer pairs taken from Kutkiene et al. 2010) which anneal within conserved gene regions. Each forward and reverse oligonucleotide contained the Fluidigm‐specific common sequence tag CS1 (5′–ACACTGACGACATGGTTCTACA–[TS–For]–3′) or CS2 (5′–TACGGTAGCAGAGACTTGGTCT–[TS–Rev]–3′) to enable subsequent barcoding of the generated PCR products (Fluidigm, San Francisco, CA, USA). PCRs and metabarcoding of *Sarcocystis*‐positive sample pools (roe deer: *n*_WT_ = 21, *n*_CA_ = 10; red deer: *n*_WT_ = 10, *n*_CA_ = 4; wild boar: *n*_WT_ = 20, *n*_CA_ = 10) were conducted as previously described (Lesniak, Franz et al., 2017).

Wolf intestinal contents were extracted and processed using the amplicon sequencing approach described in Lesniak, Heckmann et al., 2017.

### 2.4 Bioinformatics

Ungulate *Sarcocystis* sequences were sorted into operational taxonomic units (OTUs) using USEARCH (Edgar, 2010, 2013), which then were assigned to *Sarcocystis* species as described in Lesniak, Franz et al. (2017). Briefly, OTUs were assigned to *Sarcocystis* species sequences from a custom database (Lesniak, Franz et al., 2017) using BLAST^®^ (blastn; Altschul et al., 1990) with an identity threshold of 98%. Only hits with a biunique best bit score for one species were collected in a table including the respective *Sarcocystis* species, OTU, amplicon, and sample name. Due to technical limitations, the proti15_proti440_R1 and proti15_proti440_R2 datasets were split by ungulate species. When describing and discussing our results, the term “species” instead of “OTUs” will be used for simplicity, although we are aware of the technical limitations of our approach to determine species, as previously discussed (Lesniak, Heckmann et al., 2017).

The wolf metabarcoding dataset was analyzed as previously described (Lesniak, Heckmann et al., 2017).

### 2.5 Statistical analyses

The data on *Sarcocystis* presence in ungulate tissues collected using light microscopy were used to test the prediction that ungulate *Sarcocystis* prevalence was higher in areas affected by wolf recolonization. Generalized linear models (GLMs) were fitted separately for each ungulate species with binomially distributed errors, in which the response variable was the record of “*Sarcocystis* spp. infection” (binary: infected or not infected). All models included the predictors “wolf presence” (binary: absent or present) and ungulate age (categorical: juveniles, subadults, adults). To scale the *Sarcocystis* infection risk of ungulates depending on the study site, we calculated the odds ratios for each ungulate species as an exponential function of the coefficient “wolf presence” extracted from the respective GLM.

In order to interpret the goodness of fit of each model in comparison with the null model, overall likelihood ratio tests were performed with the R package *lmtest* v0.9‐34 (Zeileis & Hothorn, 2002), and model predictors were tested for collinearity using the R package *car* v2.6‐26 (Fox & Weisberg, 2011).

**Figure 1:**
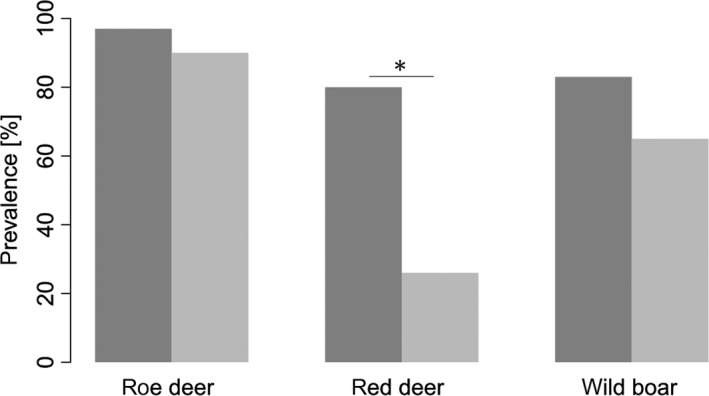
Observed *Sarcocystis* sp. prevalence in three ungulate prey species in relation to the wolf presence in their habitat. *Sarcocystis* sp. prevalence was significantly higher in wolf areas (dark gray) than in control areas (light gray) in red deer (*n*_WT_ = 75, *n*_CA_ = 18, *p* < .001), corresponding to a 47.0 times increased *Sarcocystis* sp. infection risk in red deer from the wolf area. A nonsignificant trend was found for roe deer (*n*_WT_ = 99, *n*_CA_ = 72, *p* = .075) and wild boar (*n*_WT_ = 83, *n*_CA_ = 37, *p* = .097), corresponding to a 3.8 times and 2.2 times increased infection risk of roe deer and wild boar

**Figure 2:**
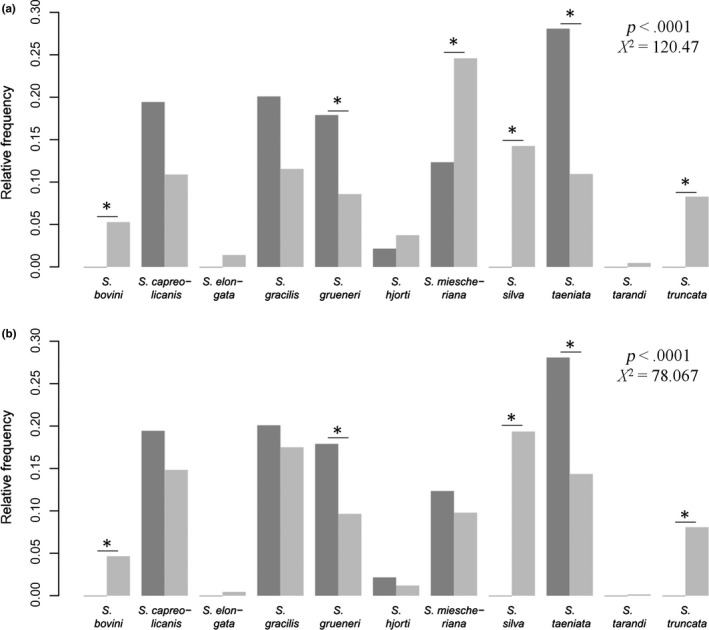
(a) Relative observed (dark gray) versus relative expected (light gray) *Sarcocystis* spp. infection frequencies in wolves based on approach A. Expected values were generated by counting genetically determined *Sarcocystis* spp. occurrences in red deer, roe deer, and wild boar and by accounting for relative, normalized ungulate feeding proportions by wolves extracted from the literature (Wagner et al., 2012). The general distribution of infection probabilities was significantly different between observed and expected values (chi‐squared test for given probabilities, χ^2^ = 120.47, *df* = 10, *p* < .0001). Binomial post hoc tests showed that *Sarcocystis grueneri* (*p* = .018) and *Sarcocystis taeniata* (*p* = .018) were overrepresented in wolves, whereas *Sarcocystis bovini* (*p* = .036), *Sarcocystis miescheriana* (*p* = .018)*, Sarcocystis silva* (*p* = .018), and *Sarcocystis truncata* (*p* = .018) were underrepresented. (b) Relative observed (dark gray) versus relative expected (light gray) *Sarcocystis* spp. infection frequencies in wolves based on approach B. Expected values have been generated by counting genetically determined *Sarcocystis* spp. occurrences in red deer, roe deer, and wild boar and by accounting for estimated relative ungulate feeding frequencies by wolves. The general distribution of infection probabilities is significantly different between observed and expected values (chi‐squared test for given probabilities, χ^2^ = 78.067, *df* = 10, *p* < .0001). Binomial post hoc tests showed that *S. grueneri* (*p* = .036) and *S. taeniata* (*p* = .018) were overrepresented in wolves, whereas *S. bovini* (*p* = .036), *S. silva* (*p* = .018) and *S. truncata* (*p* = .018) were underrepresented

In order to test whether some parasites occurred more frequently in wolves than expected, considering the frequencies of these parasites in the prey species, we first estimated expected frequencies in wolves (*f*_*exp*_), taking into account that prey species are not necessarily consumed in equal proportions. The expected frequencies were then compared to observed parasite frequencies (*f*_*obs*_) from wolves collected in this study (Figure 2a) to (1) test whether a “mismatch” could be detected in terms of a significant difference in both distributions, and (2) identify which *Sarcocystis* spp., if any, were overrepresented in wolves.

Two approaches (A and B) were used to estimate expected parasite frequencies *f*_*exp*_ in wolves. In approach A, the conventional approach, *f*_*exp*_ was estimated based on the published information on wolf diet (Wagner et al., 2012) to derive the proportion of each prey species in the diet (feeding proportion *d*_*j*_) and information on relative parasite infection frequencies *p*_*i,j*_ in ungulates obtained in this study. The observed relative frequencies pi,j of each Sarcocystis species i in ungulate species j in our sample are listed in Table 1. Based on published information on wolf diet, we used the following feeding proportions *d*_*j*_: red deer: 0.22, roe deer: 0.59, and wild boar: 0.19. Expected frequencies of *Sarcocystis* species *i* in wolves were then calculated as:

(1) $$f_{\exp,i}=\sum_{j}p_{i,j}\times d_{j}$$

**Table 1:** Relative *Sarcocystis* spp. frequencies (*f*_*inf*_) in ungulates identified by metabarcoding of microscopically positive samples

*Sarcocystis* spp.	*f*_inf_ red deer	*f*_inf_ roe deer	*f*_inf_ wild boar
Sarcocystis *bovini*	0.128	0.042	0.000
Sarcocystis *capreolicanis*	0.064	0.161	0.000
*Sarcocystis elongata*	0.064	0.000	0.000
*Sarcocystis gracilis*	0.000	0.196	0.000
*Sarcocystis grueneri*	0.128	0.098	0.000
*Sarcocystis hjorti*	0.170	0.000	0.000
*Sarcocystis miescheriana*	0.085	0.063	1.000
*Sarcocystis silva*	0.085	0.210	0.000
*Sarcocystis taeniata*	0.085	0.154	0.000
*Sarcocystis tarandi*	0.021	0.000	0.000
*Sarcocystis truncata*	0.170	0.077	0.000

red deer: *n*_WT_ = 10, *n*_CA_ = 4; roe deer: *n*_WT_ = 21, *n*_CA_ = 10; wild boar: *n*_WT_ = 20, *n*_CA_ = 10.

The conventional approach has two major drawbacks. Firstly, identified mismatches between observed and expected parasite infections in wolves could be an artifact of erroneously estimated feeding proportions *d*_*j*._ Published information on the average wolf diet is not necessarily an accurate representation of individuals in this study, and therefore *d*_*j*_ estimated in this way might be a poor representation of the diet of the wolves we analyzed. Secondly, it accounts for neither the number of preyed individuals nor the individual degree of prey parasite infestation.

To account for this potential problem, we also used a conservative approach (B) that aimed to minimize the chance of obtaining an erroneously elevated mismatch between observed and expected parasite frequencies in wolves. For this purpose, we indirectly estimated a theoretical *d*_*j*_ from the observed *Sarcocystis* frequencies in wolves and the observed *Sarcocystis* frequencies of *Sarcocystis*‐positive prey individuals. Using an optimization approach (*optim* function in R), the estimated *d*_*j*_ were those that generate expected parasite infection frequencies in Equation (1) that maximize the match between expected and observed parasite frequencies (which should minimize the risk of obtaining an erroneously elevated mismatch). Using the results from the optimization approach, the match between estimated and observed parasite infection frequencies was compared with the χ^2^ value of a chi‐squared test. As a result of this estimation, we obtained the following theoretical proportions of wolf diet composition *d*_*j*_ for each prey species: 0.09 red deer, 0.87 roe deer, and 0.04 wild boar. These estimates were then used to calculate expected parasite frequencies using Equation (1) (Figure2b).

The expected wolf *Sarcocystis* infection frequencies obtained from both approaches were used in two separate chi‐squared tests in order to check whether observed *Sarcocystis* spp. infection frequencies in wolves differed from expected probabilities. Subsequent post hoc binomial tests were used to identify *Sarcocystis* species that were overrepresented or underrepresented in wolves and their *p*‐values adjusted by applying the Benjamini–Hochberg procedure with a false discovery rate of 5% (Benjamini & Hochberg, 1995). Overrepresented *Sarcocystis* species were considered candidate species for “wolf specialists,” whereas all other species were termed “non‐wolf specialists.” Using a Mann–Whitney *U* test, we tested for each ungulate prey species whether “wolf‐specialized” *Sarcocystis* had a higher increase in prevalence (δ_prevalence_) in wolf areas relative to control areas in comparison with other detected *Sarcocystis* spp.

The wolf metabarcoding dataset was used to test the prediction that young wolves had a higher *Sarcocystis* species richness than adults, and that “wolf‐specialized” *Sarcocystis* species persist in the adult age class, whereas “nonwolf specialists” fade out. Using a GLM with Poisson‐distributed errors, we investigated whether wolf *Sarcocystis* species richness (number of species, range 0–10) decreases with wolf age (categorical: pup, yearling, adult), while controlling for wolf population size (range 3–31). The Central European lowland wolf population has been increasing exponentially from a total of three German packs in 2007 to 31 recorded packs in Germany in 2014. Post hoc tests between age categories were performed using the R package *multcomp* v1.4–5 (Hothorn, Bretz, & Westfall, 2008). In a next step, we tested whether potential “wolf‐specialized” *Sarcocystis* are more likely to persist with increasing wolf age than “non‐wolf specialists” that should be cleared by immune response. To avoid multiple testing, we only applied this test to those wolf age categories identified as age categories showing a significant decrease in *Sarcocystis* species richness. Firstly, we calculated an expected value for the average prevalence change in “wolf specialist” *Sarcocystis*. Secondly, we used a one‐sample *t* test to investigate whether this expected value deviated from the prevalence change in other species. We restricted this test to the three most common “nonspecialist” parasites that reached a minimum prevalence of 20% in wolves and that were detected in both wolves and wild ungulates.

Statistical analyses were performed in the statistical software R version 3.2.1 (R–Development–Core–Team, 2008).

## 3 RESULTS

### 3.1 Ungulate *Sarcocystis* spp. infection status and prevalence

Microscopic examination revealed that *Sarcocystis* sp. prevalence in ungulates was consistently higher in wolf areas than in control areas (Figure 1). The increase in *Sarcocystis* sp. prevalence in red deer was significant (GLM_red deer_: *p* < .001; overall likelihood ratio test: χ^2^ = 59.94, *df* = 4, *n* = 93, *p* < .001). For roe deer and wild boar, there was a nonsignificant trend of an increase in prevalence in ungulates in the wolf‐inhabited areas (GLM_roe deer_: *p* = .075; overall likelihood ratio test: χ^2^ = 19.903, *df* = 4, *n* = 171, *p* < .001; GLM_wild boar_: *p* = .097; overall likelihood ratio test: χ^2^ = 9.224, *df* = 4, *n* = 120, *p* = .024). Based on these GLMs, the odds ratios of becoming infected are 3.8 times higher for roe deer, 47.0 times higher for red deer, and 2.2 times higher for wild boar when these ungulate species originate from areas where wolves are present compared to areas where wolves are absent.

### 3.2 Ungulate and wolf *Sarcocystis* communities

A metabarcoding approach was used to determine the *Sarcocystis* species spectrum in red deer, roe deer, and wild boar. For these ungulate hosts, 148 OTUs were assigned (1–66 OTUs/*Sarcocystis* species, mean = 14) which shared the highest sequence similarity with 11 *Sarcocystis* species, and 26 OTUs were identified which were considered to be undetermined *Sarcocystis* sp. (Table S1). Based on GenBank entries, the OTUs were assigned to *Sarcocystis bovini*,* Sarcocystis capreolicanis*,* Sarcocystis grueneri*,* Sarcocystis miescheriana*,* Sarcocystis silva*,* Sarcocystis taeniata*, and *Sarcocystis truncata* in red deer and roe deer, *Sarcocystis elongata*,* Sarcocystis hjorti* and *Sarcocystis tarandi* were exclusively detected in red deer, and *Sarcocystis gracilis* only occurred in roe deer (Table S1). Wild boars were only infected with *Sarcocystis miescheriana*.

In order to test which of the detected *Sarcocystis* spp. were “wolf specialists”, two approaches were used to calculate expected *Sarcocystis* frequencies in wolves. Under the conventional more lenient approach A, the observed *Sarcocystis* spp. infection frequencies in wolves differed from the expected probabilities for several *Sarcocystis* species (χ^2^ = 120.47, *df* = 10, *p* < .0001). Post hoc tests showed that *S. grueneri* and *S. taeniata* occurred significantly more often in wolves than expected (representing potential “wolf specialists”), whereas *S. bovini*,* S. miescheriana*,* S. silva,* and *S. truncata* occurred less often than expected (Figure 2a).

Under the more conservative approach B, the observed *Sarcocystis* spp. infection frequencies in wolves also differed from expected probabilities (χ^2^ = 78.067, *df* = 10, *p* < .0001). Post hoc tests showed that the same species, *S. grueneri* and *S. taeniata*, were overrepresented in wolves, suggesting they were “wolf specialists,” whereas *S. bovini*,* S. silva,* and *S. truncata* occurred significantly less often than expected (Figure 2b).

We also tested in ungulates whether the increase in prevalence from control areas to wolf‐inhabited areas of “wolf specialist” *Sarcocystis* was higher than the increase in the other *Sarcocystis* species. Consistent with the idea of *S. grueneri* and *S. taeniata* being “wolf specialists” identified by approach A and B, their increase in prevalence from control area to wolf‐inhabited area was significantly higher than the increase in the other *Sarcocystis* species in both red deer and roe deer (red deer: *U* = 18, *p* = .044; roe deer: *U* = 18, *p* = .043).

### 3.3 *Sarcocystis* spp. species richness in wolves and prevalence of “wolf specialists”

**Figure 3:**
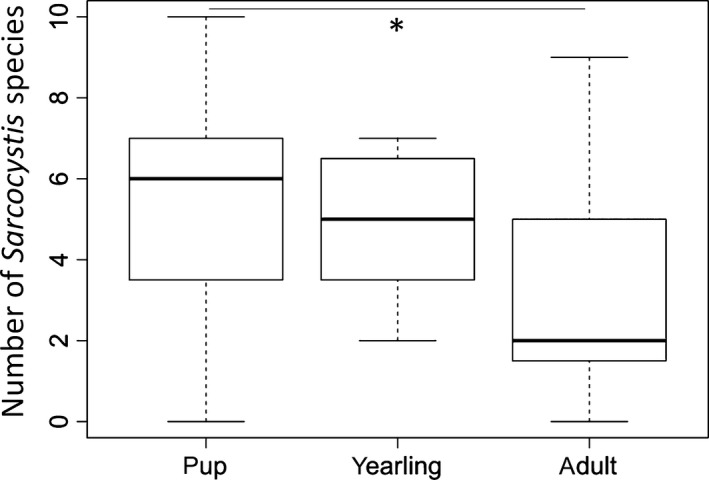
*Sarcocystis* species richness decreases with wolf age (*n*_wolves_ = 43). Pups had a significantly higher *Sarcocystis* species richness than adults (*p* = .021)

Eleven known *Sarcocystis* species were detected from wolf intestinal samples of which six were also detected in wild ungulates. Species richness per individual wolf ranged between zero and 10 species. It significantly decreased with age (GLM, overall likelihood ratio test: χ^2^ = 7.840, *df* = 3, *p* = .049, *n* = 43), and was significantly lower in adults than in pups (Tukey's post hoc test, *p* = .021, Figure 3).

**Figure 4:**
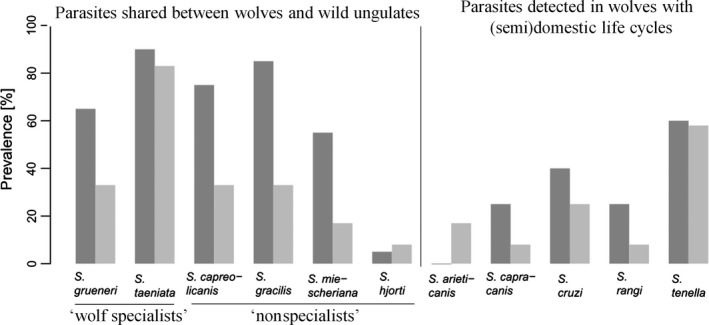
*Sarcocystis* spp. prevalence in wolf pups (*n* = 20, dark gray) and adults (*n* = 12, light gray). Eleven *Sarcocystis* species were detected in wolf intestinal samples, of which six species are shared with their wild ungulate prey species, including the “wolf specialists” *Sarcocystis taeniata* and *Sarcocystis grueneri*, whereas the other parasite species are known to have a (semi)domestic life cycle and did not occur in the investigated wild ungulates in this study. “Wolf specialists” had a lesser decrease in prevalence from pups to adults relative to “nonspecialist” *Sarcocystis* spp. (one‐sample *t* test, *t* = 5.885, C.L. 6.587–42.413, *p* = .028)

The mean value for the prevalence change of the “wolf specialists” *S. grueneri* and *S. taeniata* between wolf pups and adults was 19.5%. This expected value differed significantly from the prevalence difference of the three common nonspecialist species *S. capreolicanis*,* S. gracilis,* and *S. miescheriana* (one‐sample *t* test, *t* = 5.885, C.L. 6.587–42.413, *p* = .028, Figure 4).

## 4 DISCUSSION

In this study, we investigated whether the return of an apex predator affected parasite transmission dynamics in its ungulate prey, as measured by the prevalence of the apicomplexan genus *Sarcocystis*. For most species, sarcocysts reside in muscles in the prey and is only transferred to the definitive host if the intermediate host is eaten by a susceptible definitive host. Apicomplexans such as *Sarcocystis* with a two‐host life cycle are rarely studied in their definitive hosts due to methodological challenges (Lesniak, Heckmann et al., 2017; Moré et al., 2016; Xiang et al., 2009). In intermediate hosts, morphological cyst characteristics have frequently been used to microscopically identify *Sarcocystis* species (Malakauskas & Grikienienė, 2002; Odening, Stolte, Walter, & Bockhardt, 1995). However, oocysts or sporocysts isolated from definitive host intestinal samples do not permit morphological discrimination of species (Khan & Evans, 2006; Stronen, Sallows, Forbes, Wagner, & Paquet, 2011). To overcome such limitations, we used a combination of classical microscopy and metabarcoding to investigate *Sarcocystis* fauna, distribution, and transmission dynamics between wolves and their ungulate prey. We documented that prevalence was higher in ungulate prey in wolf‐inhabited areas than in control areas, identified *S. grueneri* and *S. taeniata* as “wolf specialists”, and showed that *Sarcocystis* species richness in wolves declined with age, whereas well‐adapted species persisted in adults.

### 4.1 *Sarcocystis* infection in ungulates

The presence of wolves in our study site was associated with a general increase in *Sarcocystis* sp. prevalence in their prey. Specifically, red deer had a much higher *Sarcocystis* sp. prevalence when sharing their habitat with wolves than animals from the control area. A similar, albeit statistically insignificant, trend was observed in roe deer and wild boar. A study from the Baltic states, where wolves have been continuously present (Chapron et al., 2014), reported similarly high prevalences of between 84.2% and 89.1% in three ungulate species, including red deer (Malakauskas & Grikienienė, 2002). The wolf‐associated prevalence differences measured in red deer in this study and the comparison to wolf range states show that wolves should be considered a more frequent and important apex predator and consumer of red deer and its sarcocysts than smaller carnivores such as red foxes (*Vulpes vulpes*) or other mesopredators. As a result, red deer‐associated *Sarcocystis* spp. cycles have increased in amplitude (prevalence and individual parasite burden) after wolf recolonization, consistent with the “fading out” hypothesis. Nevertheless, there are *Sarcocystis* spp. which can also be spread by mesopredators as definitive hosts (Moré et al., 2016), as indicated by a high sarcocysts prevalence in wild boar and roe deer in the absence of wolves. During the late 1970s, a study of roe deer in Germany described a *Sarcocystis* prevalence of 71.8%, even though no wolves were present in Central Europe at that time, which is consistent with the idea that mesopredators maintain *Sarcocystis* life cycles, as of species that infect roe deer (Entzeroth, 1981).

Another explanation for the effect of wolves on parasites in red deer and the apparent absence of a similarly strong effect in roe deer could be that both cervids differ in their regional distribution. Recolonizing wolves excreting *Sarcocystis* oocysts with their feces could now be bridging a rather patchy distribution of roe and red deer, with little overlap in terms of co‐occurrence in the same habitat in eastern Germany. There are currently no data on the distribution patterns of these ungulates from the two study regions in Germany, so it is unclear whether this is actually the case. Personal observations on hunting bags showed that usually one cervid species dominated the hunting bag when samples were collected in a particular area, consistent with findings from a study on deer densities in Scottish forests (Latham, Staines, & Gorman, 1997). Here, high red deer densities had a significant negative influence on roe deer densities. A recent study by Wu and colleagues showed that suitable habitats for red and roe deer do not necessarily overlap (Wu, Li, & Hu, 2016), even though it is generally accepted that they are sympatric species.

### 4.2 Comparison of *Sarcocystis* communities between intermediate (ungulate) and definitive (wolf) hosts

In *Sarcocystis* life cycles, several intermediate and definitive hosts can be involved which may be linked with each other through the food web. In this study, two species, *S. grueneri* and *S. taeniata*, appear to be well adapted to wolves as definitive hosts. Both species occurred in wolves more often than expected on the basis of parasite distribution in prey species. For both red deer and roe deer, they showed the strongest increases in prevalence in wolf areas compared to other *Sarcocystis* species. Only red deer from wolf areas were infected with these two types of *Sarcocystis* spp., although some roe deer from the control site also hosted *S. grueneri* and *S. taeniata*. These findings suggest that (1) *S. grueneri* and *S. taeniata* spread in wolf areas are “wolf specialists,” and that (2) other potential canid definitive hosts such as domestic dogs (*Canis lupus familiaris*), red foxes or raccoon dogs (*Nyctereutes procyonoides*) spread other strains of *S. grueneri* and *S. taeniata* in the absence of wolves. *Sarcocystis* screening of other definitive hosts using metabarcoding techniques will be necessary to clarify the epidemiological relationships among intermediate and additional definitive hosts of this protozoan.

### 4.3 Age‐related *Sarcocystis* infection in wolves

Experimental approaches in *Toxoplasma* serve as a model to understand the immunological response of hosts in their interaction with parasites in protozoan infections (Leng, Butcher, & Denkers, 2009). These studies mainly focus on the intermediate hosts, as these are more severely affected by the disease than the definitive hosts which usually suffer little mortality and diarrhea at worst (Di Genova & Tonelli, 2016; Liang, Granstrom, Zhao, & Timoney, 1998). In definitive hosts, studies of immunological defense mechanisms toward apicomplexan parasites have often focused on mouse models (Di Genova & Tonelli, 2016) or, in the well‐studied case of *Toxoplasma*, feline species (Rush, Lappin, & Milhausen, 2001).

Even though it is not clear which molecular mechanisms are responsible for *Sarcocystis* defense in wolves, wolf pups hosted more *Sarcocystis* species than older animals, consistent with the predictions from the “fading out” hypothesis that immunological resistance to *Sarcocystis* is higher in adults. This finding is also consistent with studies of domestic dogs where *Cystoisospora* and *Giardia* infections were most prevalent in pups (Barutzki & Schaper, 2003; Bugg, Robertson, Elliot, & Thompson, 1999). In wild canids, comparable indications of age‐related parasite burden have previously only been reported in helminth etiopathology (Guberti, Stancampiano, & Francisci, 1993; Lesniak, Heckmann et al., 2017; Veronesi et al., 2014; Webster et al., 2017). In this study, we investigated this phenomenon accepting that coevolution in a host–parasite arms race can drive hosts to counteract parasite invasion by developing immune defense mechanisms. In turn, parasites are expected to evolve strategies to circumvent such barriers (Dawkins & Krebs, 1979; Schmid‐Hempel, 2011), which can result in a higher persistence of such parasites despite an increasing immune competence of maturing host individuals. *Sarcocystis grueneri* and *S. taeniata* appear to be particularly well adapted to wolves as indicated by their lower decrease in prevalence during wolf maturation compared to other *Sarcocystis* spp. If these parasites have adapted to wolves, it may represent a more subtle adaptation than immune escape and might benefit from further investigation.

### 4.4 Ungulate *Sarcocystis* spp. fauna

The *Sarcocystis* fauna in red deer has been thoroughly studied with 10 species described so far. Of these, seven species were found in our red deer samples: *S. capreolicanis* (Wesemeier & Sedlaczek, 1995b), *S. grueneri* (Prakas & Butkauskas, 2012; Wesemeier & Sedlaczek, 1995b), *S. elongata* (Gjerde, 2014b), *S. hjorti* (Dahlgren & Gjerde, 2010; Gjerde, 2013), *S. taeniata* (Reissig, Moré, Massone, & Uzal, 2016), *S. tarandi* (Dahlgren & Gjerde, 2010; Gjerde, 2014b), and *S. truncata* (Gjerde, 2014b) (Figure S2b). *S. hardangeri*,* S. ovalis,* and *S. rangiferi* (Dahlgren & Gjerde, 2010) were previously isolated in Norwegian hosts and not found in this study. This is the first study to document *S. silva*—previously only known from roe deer and moose (*Alces alces*)—and the recently characterized species *S. bovini* from German ungulates (Gjerde, 2016) as well as the supposedly suid‐specific *S. miescheriana* (Coelho et al., 2015) for the first time in red deer.

Previous molecular studies investigated the *Sarcocystis* species composition of roe deer, yielding four genetically characterized species. Only *S. oviformis* (Gjerde, 2012; Kolenda et al., 2014) was not found in our study whereas *S. capreolicanis* (Gjerde, 2012; Prakas & Butkauskas, 2012), *S. gracilis* (Gjerde, 2012; Kolenda et al., 2014), and *S. silva* (Gjerde, 2012; Kolenda et al., 2014) were found. This is also the first record of *S. bovini*,* S. grueneri*,* S. miescheriana, S. taeniata*, and *S. truncata* in this ungulate (Figure S2a). *Sarcocystis bovini* was previously only described from cattle (Gjerde, 2016). *Sarcocystis grueneri* was described from reindeer (*Rangifer tarandus*) (Gjerde, 1986), fallow deer (*Dama dama*) (Wesemeier & Sedlaczek, 1995a), and red deer (Prakas & Butkauskas, 2012; Wesemeier & Sedlaczek, 1995b). *Sarcocystis taeniata* was identified in sika deer (*Cervus nippon*) (Prakas et al., 2016), red deer (Reissig et al., 2016), and moose (Gjerde, 2014a). *Sarcocystis truncata* was previously only described from red deer (Gjerde, 2014b).

In wild boar, one of two known *Sarcocystis* species was identified. We confirmed *S. miescheriana* (Kia, Mirhendi, Rezaeian, Zahabiun, & Sharbatkhori, 2011; Prakas & Butkauskas, 2012) but not the zoonotic *S. suihominis* (Prakas & Butkauskas, 2012) in our sample of wild boar (Figure S2c). The fact that the supposedly suid‐specific species *S. miescheriana* was also detected in cervids, and that *S. miescheriana* reads sequenced in this study had a higher intraspecific diversity than all other detected sarcocystis (Table S1), suggests that *S. miescheriana* sequences deposited in GenBank could potentially derive from more than the one species characterized to date.

## CONFLICT OF INTEREST

None declared.

## AUTHOR CONTRIBUTIONS

This study was designed by HH, IL, and OK; IL and OK collected and dissected the carcasses; IL performed the microscopic analysis; EH and IL performed the molecular analysis; IH and IL did the bioinformatic data analysis; HH, IL, and MF performed the statistical analyses; IL wrote the manuscript with contributions of ADG, HH, and MF. All authors contributed critically to the drafts and gave final approval for publication.

## DATA ACCESSIBILITY

Data used in this publication and corresponding description files are available under the following DOI: https://doi.org/10.5061/dryad.sk435.

## Supporting information


** **

## References

[ece33839-bib-0001] Andersen, L. W. , Harms, V. , Caniglia, R. , Czarnomska, S. D. , Fabbri, E. , Jedrzejewska, B. , … Stronen, A. V. (2015). Long‐distance dispersal of a wolf, *Canis lupus*, in northwestern Europe. Mammal Research, 60, 163–168. https://doi.org/10.1007/s13364-015-0220-6

[ece33839-bib-0002] Ansorge, H. , Holzapfel, M. , Kluth, G. , Reinhardt, I. , & Wagner, C. (2010). Die Rückkehr der Wölfe. Das erste Jahrzehnt. Biologie in unserer Zeit, 40, 244–253. https://doi.org/10.1002/biuz.201010425

[ece33839-bib-0500] Altschul, S. F. , Gish, W. , Miller, W. , Myers, E. W. & Lipman, D. J. (1990). Basic local alignment search tool. J Mol Biol, 215, 403–410. 2231712 10.1016/S0022-2836(05)80360-2

[ece33839-bib-0003] Barutzki, D. , & Schaper, R. (2003). Endoparasites in dogs and cats in Germany 1999‐2002. Parasitology Research, 90(Suppl 3), S148–S150. https://doi.org/10.1007/s00436-003-0922-6 12928886 10.1007/s00436-003-0922-6

[ece33839-bib-0004] Benjamini, Y. , & Hochberg, Y. (1995). Controlling the false discovery rate: A practical and powerful approach to multiple testing. Journal of the Royal Statistical Society. Series B (Methodological), 57, 289–300.

[ece33839-bib-0005] Bugg, R. J. , Robertson, I. D. , Elliot, A. D. , & Thompson, R. C. (1999). Gastrointestinal parasites of urban dogs in Perth, Western Australia. The Veterinary Journal, 157, 295–301. https://doi.org/10.1053/tvjl.1998.0327 10328840 10.1053/tvjl.1998.0327

[ece33839-bib-0006] Buxton, D. (1998). Protozoan infections (*Toxoplasma gondii*,* Neospora caninum* and *Sarcocystis* spp.) in sheep and goats: Recent advances. Veterinary Research, 29, 289–310. 9689743

[ece33839-bib-0007] Chapron, G. , Kaczensky, P. , Linnell, J. D. , von Arx, M. , Huber, D. , Andren, H. , … Boitani, L. (2014). Recovery of large carnivores in Europe's modern human‐dominated landscapes. Science, 346, 1517–1519. https://doi.org/10.1126/science.1257553 25525247 10.1126/science.1257553

[ece33839-bib-0008] Coelho, C. , Gomes, J. , Inácio, J. , Amaro, A. , Mesquita, J. R. , Pires, I. , … Vieira‐Pinto, M. (2015). Unraveling *Sarcocystis miescheriana* and *Sarcocystis suihominis* infections in wild boar. Veterinary Parasitology, 212, 100–104. https://doi.org/10.1016/j.vetpar.2015.08.015 26319199 10.1016/j.vetpar.2015.08.015

[ece33839-bib-0009] Craig, H. L. , & Craig, P. S. (2005). Helminth parasites of wolves (*Canis lupus*): A species list and an analysis of published prevalence studies in Nearctic and Palaearctic populations. Journal of Helminthology, 79, 95–103. https://doi.org/10.1079/JOH2005282 15946392 10.1079/joh2005282

[ece33839-bib-0010] Dahlgren, S. S. , & Gjerde, B. (2010). Molecular characterization of five *Sarcocystis* species in red deer (*Cervus elaphus*), including *Sarcocystis hjorti* n. sp., reveals that these species are not intermediate host specific. Parasitology, 137, 815–840. https://doi.org/10.1017/S0031182009991569 19961651 10.1017/S0031182009991569

[ece33839-bib-0011] Dawkins, R. , & Krebs, J. R. (1979). Arms races between and within species. Proceedings of the Royal Society of London. Series B, Biological Sciences, 205, 489–511. https://doi.org/10.1098/rspb.1979.0081 10.1098/rspb.1979.0081 42057

[ece33839-bib-0012] Di Genova, B. M. , & Tonelli, R. R. (2016). Infection strategies of intestinal parasite pathogens and host cell responses. Frontiers in Microbiology, 7, 256. 26973630 10.3389/fmicb.2016.00256 PMC4776161

[ece33839-bib-0013] Dubey, J. P. , Calero_Bernal, R. , Rosenthal, B. M. , Speer, C. A. , & Fayer, R. (2015). Sarcocystosis of animals and humans, 2nd ed. Boca Raton, FL: Taylor Francis Inc https://doi.org/10.1201/b19184

[ece33839-bib-0014] Dubey, J. P. , & Lindsay, D. S. (2006). Neosporosis, toxoplasmosis, and sarcocystosis in ruminants. The Veterinary Clinics of North America. Food Animal Practice, 22, 645–671. https://doi.org/10.1016/j.cvfa.2006.08.001 17071358 10.1016/j.cvfa.2006.08.001

[ece33839-bib-0015] East, M. L. , Bassano, B. , & Ytrehus, B. (2011). The role of pathogens in the population dynamics of European ungulates In Apollonio M., Andersen R. & Putman R. (Eds.), Ungulate management in Europe: Problems and practices (pp. 319–348). Cambridge, UK: Cambridge University Press https://doi.org/10.1017/CBO9780511974137

[ece33839-bib-0016] Edgar, R. C. (2010). Search and clustering orders of magnitude faster than BLAST. Bioinformatics, 26, 2460–2461. https://doi.org/10.1093/bioinformatics/btq461 20709691 10.1093/bioinformatics/btq461

[ece33839-bib-0017] Edgar, R. C. (2013). UPARSE: Highly accurate OTU sequences from microbial amplicon reads. Nature Methods, 10, 996–998. https://doi.org/10.1038/nmeth.2604 23955772 10.1038/nmeth.2604

[ece33839-bib-0018] Entzeroth, R. (1981). Experiments on Sarcosporidia of the indigenous roe deer (*Capreolus capreolus* L.). Zeitschrift fuer Jagdwissenschaft, 27, 247–257.

[ece33839-bib-0019] Estes, J. A. , Terborgh, J. , Brashares, J. S. , Power, M. E. , Berger, J. , Bond, W. J. , … Wardle, D. A. (2011). Trophic downgrading of planet Earth. Science, 333, 301–306. https://doi.org/10.1126/science.1205106 21764740 10.1126/science.1205106

[ece33839-bib-0020] Farrell, M. J. , Stephens, P. R. , Berrang‐Ford, L. , Gittleman, J. L. , & Davies, T. J. (2015). The path to host extinction can lead to loss of generalist parasites. Journal of Animal Ecology, 84, 978–984. https://doi.org/10.1111/1365-2656.12342 25640629 10.1111/1365-2656.12342

[ece33839-bib-0021] Fox, J. , & Weisberg, S. (2011). An R companion to applied regression, 2nd ed. Thousand Oaks, CA: SAGE Publications Inc.

[ece33839-bib-0022] Gjerde, B. (1986). Scanning electron microscopy of the sarcocysts of six species of *Sarcocystis* from reindeer (*Rangifer tarandus tarandus*). Acta Pathologica, Microbiologica, et Immunologica Scandinavica. Section B, Microbiology, 94, 309–317. 10.1111/j.1699-0463.1986.tb03058.x 3098040

[ece33839-bib-0023] Gjerde, B. (2012). Morphological and molecular characterization and phylogenetic placement of *Sarcocystis capreolicanis* and *Sarcocystis silva* n. sp. from roe deer (*Capreolus capreolus*) in Norway. Parasitology Research, 110, 1225–1237. https://doi.org/10.1007/s00436-011-2619-6 21853224 10.1007/s00436-011-2619-6

[ece33839-bib-0024] Gjerde, B. (2013). Phylogenetic relationships among *Sarcocystis* species in cervids, cattle and sheep inferred from the mitochondrial cytochrome c oxidase subunit I gene. International Journal for Parasitology, 43, 579–591. https://doi.org/10.1016/j.ijpara.2013.02.004 23542092 10.1016/j.ijpara.2013.02.004

[ece33839-bib-0025] Gjerde, B. (2014a). Morphological and molecular characteristics of four *Sarcocystis* spp. in Canadian moose (*Alces alces*), including *Sarcocystis taeniata* n. sp. Parasitology Research, 113, 1591–1604. https://doi.org/10.1007/s00436-014-3806-z 24535735 10.1007/s00436-014-3806-z

[ece33839-bib-0026] Gjerde, B. (2014b). *Sarcocystis* species in red deer revisited: With a re‐description of two known species as *Sarcocystis elongata* n. sp. and *Sarcocystis truncata* n. sp. based on mitochondrial cox1 sequences. Parasitology, 141, 441–452. https://doi.org/10.1017/S0031182013001819 24230915 10.1017/S0031182013001819

[ece33839-bib-0027] Gjerde, B. (2016). Molecular characterisation of *Sarcocystis bovifelis*,* Sarcocystis bovini* n. sp., *Sarcocystis hirsuta* and *Sarcocystis cruzi* from cattle (*Bos taurus*) and *Sarcocystis sinensis* from water buffaloes (*Bubalus bubalis*). Parasitology Research, 115, 1473–1492. https://doi.org/10.1007/s00436-015-4881-5 26677095 10.1007/s00436-015-4881-5

[ece33839-bib-0028] Guberti, V. , Stancampiano, L. , & Francisci, F. (1993). Intestinal helminth parasite community in wolves (*Canis lupus*) in Italy. Parassitologia, 35, 59–65. 8065823

[ece33839-bib-0029] Hothorn, T. , Bretz, F. , & Westfall, P. (2008). Simultaneous inference in general parametric models. Biometrical Journal, 50, 346–363. https://doi.org/10.1002/(ISSN)1521-4036 18481363 10.1002/bimj.200810425

[ece33839-bib-0030] Kelly, D. W. , Paterson, R. A. , Townsend, C. R. , Poulin, R. , & Tompkins, D. M. (2009). Parasite spillback: A neglected concept in invasion ecology? Ecology, 90, 2047–2056. https://doi.org/10.1890/08-1085.1 19739367 10.1890/08-1085.1

[ece33839-bib-0031] Khan, R. A. , & Evans, L. (2006). Prevalence of *Sarcocystis* spp. in two subspecies of caribou (*Rangifer tarandus*) in Newfoundland and Labrador, and foxes (*Vulpes vulpes*), wolves (*Canis lupus*), and husky dogs (*Canis familiaris*) as potential definitive hosts. Journal of Parasitology, 92, 662–663. https://doi.org/10.1645/GE-753R1.1 16884021 10.1645/GE-753R1.1

[ece33839-bib-0032] Kia, E. B. , Mirhendi, H. , Rezaeian, M. , Zahabiun, F. , & Sharbatkhori, M. (2011). First molecular identification of *Sarcocystis miescheriana* (Protozoa, Apicomplexa) from wild boar (*Sus scrofa*) in Iran. Experimental Parasitology, 127, 724–726. https://doi.org/10.1016/j.exppara.2010.11.007 21095184 10.1016/j.exppara.2010.11.007

[ece33839-bib-0033] Kolenda, R. , Ugorski, M. , & Bednarski, M. (2014). Molecular characterization of *Sarcocystis* species from Polish roe deer based on ssu rRNA and cox1 sequence analysis. Parasitology Research, 113, 3029–3039. https://doi.org/10.1007/s00436-014-3966-x 24948101 10.1007/s00436-014-3966-x PMC4110405

[ece33839-bib-0501] Kutkienė, L. , Prakas, P. , Sruoga, A. & Butkauskas, D. (2010). The mallard duck (Anas platyrhynchos) as intermediate host for Sarcocystis wobeseri sp. nov. from the barnacle goose (Branta leucopsis). Parasitol Res, 107, 879–888. 20567986 10.1007/s00436-010-1945-4

[ece33839-bib-0034] Latham, J. , Staines, B. W. , & Gorman, M. L. (1997). Correlations of red (*Cervus elaphus*) and roe (*Capreolus capreolus*) deer densities in Scottish forests with environmental variables. Journal of Zoology, 242, 681–704. https://doi.org/10.1111/j.1469-7998.1997.tb05820.x

[ece33839-bib-0035] Leng, J. , Butcher, B. A. , & Denkers, E. Y. (2009). Dysregulation of macrophage signal transduction by *Toxoplasma gondii*: Past progress and recent advances. Parasite Immunology, 31, 717–728. https://doi.org/10.1111/j.1365-3024.2009.01122.x 19891610 10.1111/j.1365-3024.2009.01122.x PMC2774889

[ece33839-bib-0036] Lesniak, I. , Franz, M. , Heckmann, I. , Greenwood, A. D. , Hofer, H. , & Krone, O. (2017). Surrogate hosts: Hunting dogs and recolonizing grey wolves share their endoparasites. International Journal for Parasitology: Parasites and Wildlife, 6, 278–286. 28951833 10.1016/j.ijppaw.2017.09.001 PMC5605491

[ece33839-bib-0037] Lesniak, I. , Heckmann, I. , Heitlinger, E. , Szentiks, C. A. , Nowak, C. , Harms, V. , … Krone, O. (2017). Population expansion and individual age affect endoparasite richness and diversity in a recolonising large carnivore population. Scientific Reports, 7, 41730 https://doi.org/10.1038/srep41730 28128348 10.1038/srep41730 PMC5269671

[ece33839-bib-0038] Liang, F. T. , Granstrom, D. E. , Zhao, X. M. , & Timoney, J. F. (1998). Evidence that surface proteins Sn14 and Sn16 of *Sarcocystis* neurona merozoites are involved in infection and immunity. Infection and Immunity, 66, 1834–1838. 9573058 10.1128/iai.66.5.1834-1838.1998 PMC108132

[ece33839-bib-0039] Malakauskas, M. , & Grikienienė, J. (2002). *Sarcocystis* infection in wild ungulates in Lithuania. Acta Zoologica Lituanica, 12, 372–380. https://doi.org/10.1080/13921657.2002.10512527

[ece33839-bib-0040] Moré, G. , Maksimov, A. , Conraths, F. J. , & Schares, G. (2016). Molecular identification of *Sarcocystis* spp. in foxes (*Vulpes vulpes*) and raccoon dogs (*Nyctereutes procyonoides*) from Germany. Veterinary Parasitology, 220, 9–14. https://doi.org/10.1016/j.vetpar.2016.02.011 26995715 10.1016/j.vetpar.2016.02.011

[ece33839-bib-0041] Munday, B. L. , Hartley, W. J. , Harrigan, K. E. , Presidente, P. J. A. , & Obendorf, D. L. (1979). S*arcocystis* and related organisms in Australian Wildlife: II. Survey findings in birds, reptiles, amphibians and fish. Journal of Wildlife Diseases, 15, 57–73. https://doi.org/10.7589/0090-3558-15.1.57 110950 10.7589/0090-3558-15.1.57

[ece33839-bib-0042] Nowak, S. , & Mysłajek, R. W. (2016). Wolf recovery and population dynamics in Western Poland, 2001–2012. Mammal Research, 61, 83–98. https://doi.org/10.1007/s13364-016-0263-3

[ece33839-bib-0043] Nowak, S. , Mysłajek, R. W. , Kłosińska, A. , & Gabryś, G. (2011). Diet and prey selection of wolves (*Canis lupus*) recolonising Western and Central Poland. Mammalian Biology‐Zeitschrift für Säugetierkunde, 76, 709–715. https://doi.org/10.1016/j.mambio.2011.06.007

[ece33839-bib-0044] Odening, K. , Stolte, M. , Walter, G. , & Bockhardt, I. (1995). Cyst wall ultrastructure of two *Sarcocystis* spp. from European Mouflon (*Ovis ammon musimon*) in Germany compared with domestic sheep. Journal of Wildlife Diseases, 31, 550–554. https://doi.org/10.7589/0090-3558-31.4.550 8592390 10.7589/0090-3558-31.4.550

[ece33839-bib-0045] Onac, D. , Győrke, A. , Oltean, M. , Gavrea, R. , & Cozma, V. (2013). First detection of *Echinococcus granulosus* G1 and G7 in wild boars (*Sus scrofa*) and red deer (*Cervus elaphus*) in Romania using PCR and PCR‐RFLP techniques. Veterinary Parasitology, 193, 289–291. https://doi.org/10.1016/j.vetpar.2012.11.044 23332123 10.1016/j.vetpar.2012.11.044

[ece33839-bib-0046] Poulsen, C. S. , & Stensvold, C. R. (2014). Current status of epidemiology and diagnosis of human sarcocystosis. Journal of Clinical Microbiology, 52, 3524–3530. https://doi.org/10.1128/JCM.00955-14 24759707 10.1128/JCM.00955-14 PMC4187749

[ece33839-bib-0047] Prakas, P. , & Butkauskas, D. (2012). Protozoan parasites from genus *Sarcocystis* and their investigations in Lithuania. Ekologija, 58, 45–58.

[ece33839-bib-0048] Prakas, P. , Butkauskas, D. , Rudaityte, E. , Kutkiene, L. , Sruoga, A. , & Puraite, I. (2016). Morphological and molecular characterization of *Sarcocystis taeniata* and *Sarcocystis pilosa* n. sp. from the sika deer (*Cervus nippon*) in Lithuania. Parasitology Research, 115, 3021–3032. https://doi.org/10.1007/s00436-016-5057-7 27086872 10.1007/s00436-016-5057-7

[ece33839-bib-0049] R–Development–Core–Team (2008). R: A language and environment for statistical computing. Retrieved from http://www.R-project.org

[ece33839-bib-0050] Reinhardt, I. , Kluth, G. , Nowak, S. , & Myslajek, R. (2015). Standards for the monitoring of the Central European wolf population in Germany and Poland. BfN Federal Agency for Nature Conservation, 398, 43.

[ece33839-bib-0051] Reissig, E. C. , Moré, G. , Massone, A. , & Uzal, F. A. (2016). Sarcocystosis in wild red deer (*Cervus elaphus*) in Patagonia, Argentina. Parasitology Research, 115, 1773–1778. https://doi.org/10.1007/s00436-016-4915-7 **,** 1‐6. 26779923 10.1007/s00436-016-4915-7

[ece33839-bib-0052] Rocchigiani, G. , Nardoni, S. , D'Ascenzi, C. , Nicoloso, S. , Picciolli, F. , Papini, R. A. , & Mancianti, F. (2016). Seroprevalence of *Toxoplasma gondii* and *Neospora caninum* in red deer from Central Italy. Annals of Agricultural and Environmental Medicine, 23, 699–701. https://doi.org/10.5604/12321966.1226870 28030947 10.5604/12321966.1226870

[ece33839-bib-0053] Rush, A. , Lappin, M. , & Milhausen, M. (2001). Analysis of the humoral responses of Toxoplasma gondii‐infected cats using immunofluorescent assays with tachyzoite, bradyzoite, and gametogenic stages. Journal of Parasitology, 87, 83–89. 11227907 10.1645/0022-3395(2001)087[0083:AOTHRO]2.0.CO;2

[ece33839-bib-0054] Schmid‐Hempel, P. (2011). Evolutionary parasitology: The integrated study of infections, immunology, ecology and genetics. Oxford, UK: Oxford University Press.

[ece33839-bib-0055] Shaapan, R. M. (2016). The common zoonotic protozoal diseases causing abortion. Journal of Parasitic Diseases, 40, 1116–1129. https://doi.org/10.1007/s12639-015-0661-5 27876900 10.1007/s12639-015-0661-5 PMC5118287

[ece33839-bib-0056] Stronen, A. V. , Sallows, T. , Forbes, G. J. , Wagner, B. , & Paquet, P. C. (2011). Diseases and parasites in wolves of the Riding Mountain National Park region, Manitoba, Canada. Journal of Wildlife Diseases, 47, 222–227. https://doi.org/10.7589/0090-3558-47.1.222 21270013 10.7589/0090-3558-47.1.222

[ece33839-bib-0057] Szentiks, C. A. , Fritsch, G. , Lesniak, I. , Galateanu, G. , Mühldorfer, K. , Hildebrandt, T. B. , … Krone, O. (2016). One decade of health monitoring in German free‐ranging wolves (Canis lupus). 12th Conference of the European Wildlife Disease Association (EWDA), pp. 81. Berlin, Germany.

[ece33839-bib-0058] Veronesi, F. , Morganti, G. , di Cesare, A. , Lepri, E. , Cassini, R. , Zanet, S. , … Ferroglio, E. (2014). *Eucoleus boehmi* infection in red fox (*Vulpes vulpes*) from Italy. Veterinary Parasitology, 206, 232–239. https://doi.org/10.1016/j.vetpar.2014.10.001 25458564 10.1016/j.vetpar.2014.10.001

[ece33839-bib-0059] Wagner, C. , Holzapfel, M. , Kluth, G. , Reinhardt, I. , & Ansorge, H. (2012). Wolf (*Canis lupus*) feeding habits during the first eight years of its occurrence in Germany. Mammalian Biology‐Zeitschrift für Säugetierkunde, 77, 196–203. https://doi.org/10.1016/j.mambio.2011.12.004

[ece33839-bib-0060] Webster, P. , Monrad, J. , Kapel, C. M. O. , Kristensen, A. T. , Jensen, A. L. , & Thamsborg, S. M. (2017). The effect of host age and inoculation dose on infection dynamics of *Angiostrongylus vasorum* in red foxes (*Vulpes vulpes*). Parasites & Vectors, 10, 4 https://doi.org/10.1186/s13071-016-1940-4 28049507 10.1186/s13071-016-1940-4 PMC5209822

[ece33839-bib-0061] Wesemeier, H. H. , & Sedlaczek, J. (1995a). One known *Sarcocystis* species and one found for the first time in fallow deer (*Dama dama*). Applied Parasitology, 36, 299–302. 8528305

[ece33839-bib-0062] Wesemeier, H. H. , & Sedlaczek, J. (1995b). One known *Sarcocystis* species and two found for the first time in red deer and wapiti (*Cervus elaphus*) in Europe. Applied Parasitology, 36, 245–251. 8528304

[ece33839-bib-0063] Wu, W. , Li, Y. , & Hu, Y. (2016). Simulation of potential habitat overlap between red deer (*Cervus elaphus*) and roe deer (*Capreolus capreolus*) in northeastern China. PeerJ, 4, e1756 https://doi.org/10.7717/peerj.1756 27019775 10.7717/peerj.1756 PMC4806631

[ece33839-bib-0064] Xiang, Z. , Chen, X. , Yang, L. , He, Y. , Jiang, R. , Rosenthal, B. M. , … Yang, Z. (2009). Non‐invasive methods for identifying oocysts of *Sarcocystis* spp. from definitive hosts. Parasitology International, 58, 293–296. https://doi.org/10.1016/j.parint.2009.03.004 19336258 10.1016/j.parint.2009.03.004

[ece33839-bib-0065] Zeileis, A. , & Hothorn, T. (2002). Diagnostic checking in regression relationships. R News, 2, 7–10.

