# Supplementary material for: Recolonizing gray wolves increase parasite infection risk in their prey
Source: Ecol Evol. 2018 Jan 22;8(4):2160–70. doi: 10.1002/ece3.3839 (PMC5817143; doi:10.1002/ece3.3839)
Supplement: Supplementary file 1 [file ECE3-8-2160-s001.pdf]

# Supplementary Information

# Recolonizing grey wolves increase parasite infection risk in their prey

Ines Lesniak[1,*], Ilja Heckmann[1], Mathias Franz[1], Alex D. Greenwood[1,2], Emanuel Heitlinger[1,4], Heribert Hofer[1,2,3,†], Oliver Krone[1,†]

[1]Leibniz Institute for Zoo and Wildlife Research, Alfred–Kowalke–Straße 17, 10315 Berlin, Germany

[2]Department of Veterinary Medicine, Freie Universität Berlin, Oertzenweg 19b, 14163 Berlin, Germany

[3]Department of Biology, Chemistry, Pharmacy, Freie Universität Berlin, Takustraße 3, 14195 Berlin, Germany

[4]Humboldt–Universität zu Berlin, Ecology and Evolution of Molecular Parasite Host Interactions, Philippstraße 13, 10115 Berlin, Germany

[*]corresponding author: Ines Lesniak, lesniak@izw-berlin.de

[†] both authors contributed equally

**Suppl. Table S1:** Number of OTUs per ungulate *Sarcocystis* species (mean = 14 OTUs/species) based on 18S rRNA sequences from 10 different amplicons analysed with USEARCH and number of all and unique 18S rRNA GenBank entries for each species.

| species assignment | $n_{OTUs}$ | n 18S rRNA GenBank entries | n unique 18S rRNA GenBank entries |
|---|---|---|---|
| *S. bovini* | 8 | 26 | 26 |
| *S. capreolicanis* | 9 | 12 | 12 |
| *S. elongata* | 2 | 20 | 18 |
| *S. gracilis* | 5 | 14 | 7 |
| *S. grueneri* | 3 | 2 | 2 |
| *S. hjorti* | 5 | 6 | 4 |
| *S. miescheriana* | 66 | 49 | 26 |
| *S. silva* | 18 | 9 | 8 |
| *Sarcocystis* sp. | 26 | NA | NA |
| *S. taeniata* | 27 | 27 | 26 |
| *S. tarandi* | 1 | 22 | 22 |
| *S. truncata* | 4 | 20 | 20 |

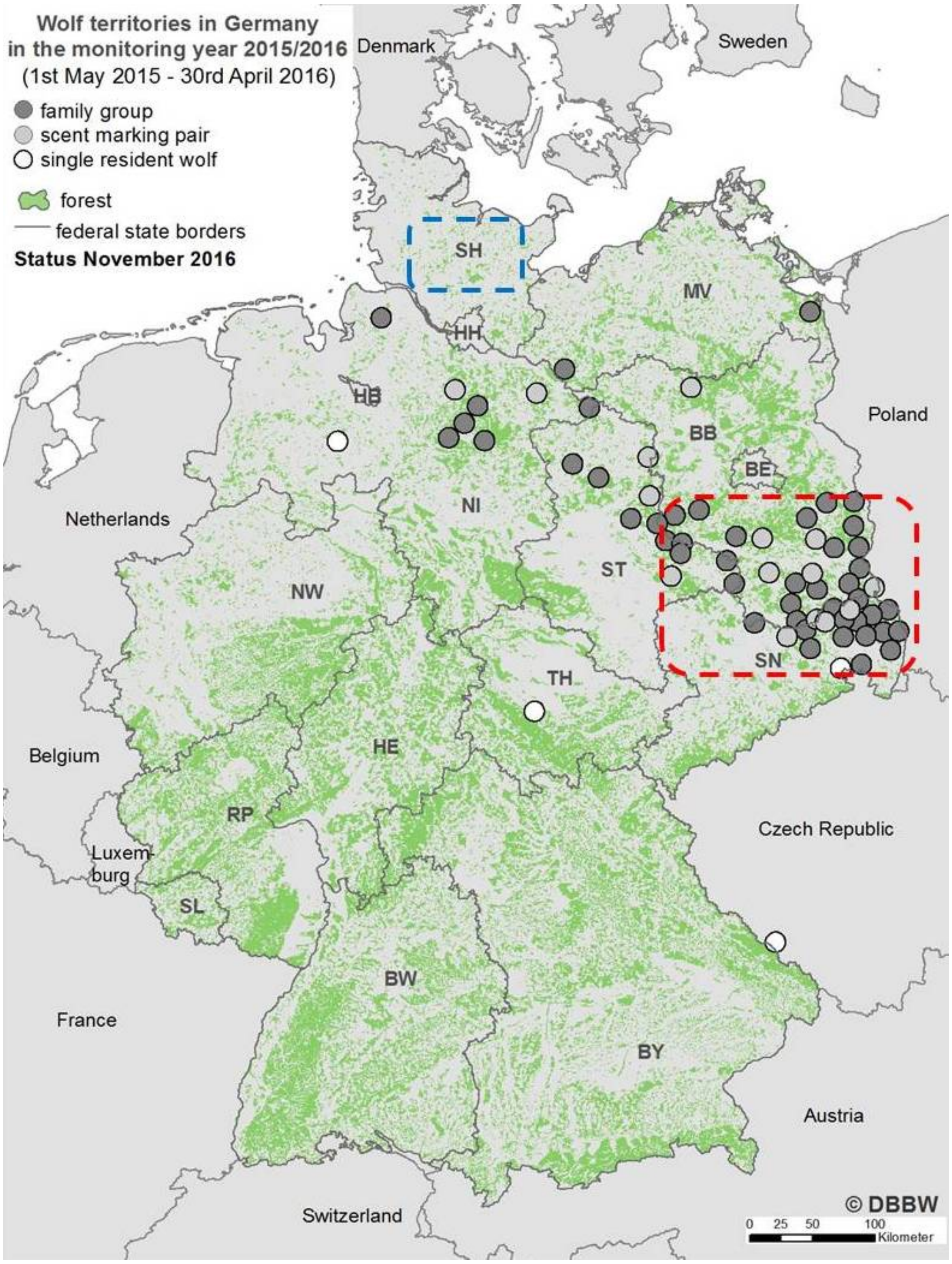


**Suppl. Figure 1:** Distribution of grey wolves in Germany and ungulate sampling sites. First wolf packs recolonized the region Lusatia along the federal state borders of Brandenburg (BB) and Saxony (SN) in the year 2000, from where wolves expanded their range into northwesterly direction. Until today, no resident wolves have been documented in the federal state of Schleswig–Holstein (SH). The blue ('control site' without wolves) and red ('wolf area') dotted lines frame the study sites where ungulates were collected. Figure courtesy of the German Wolf Documentation and Information Center.

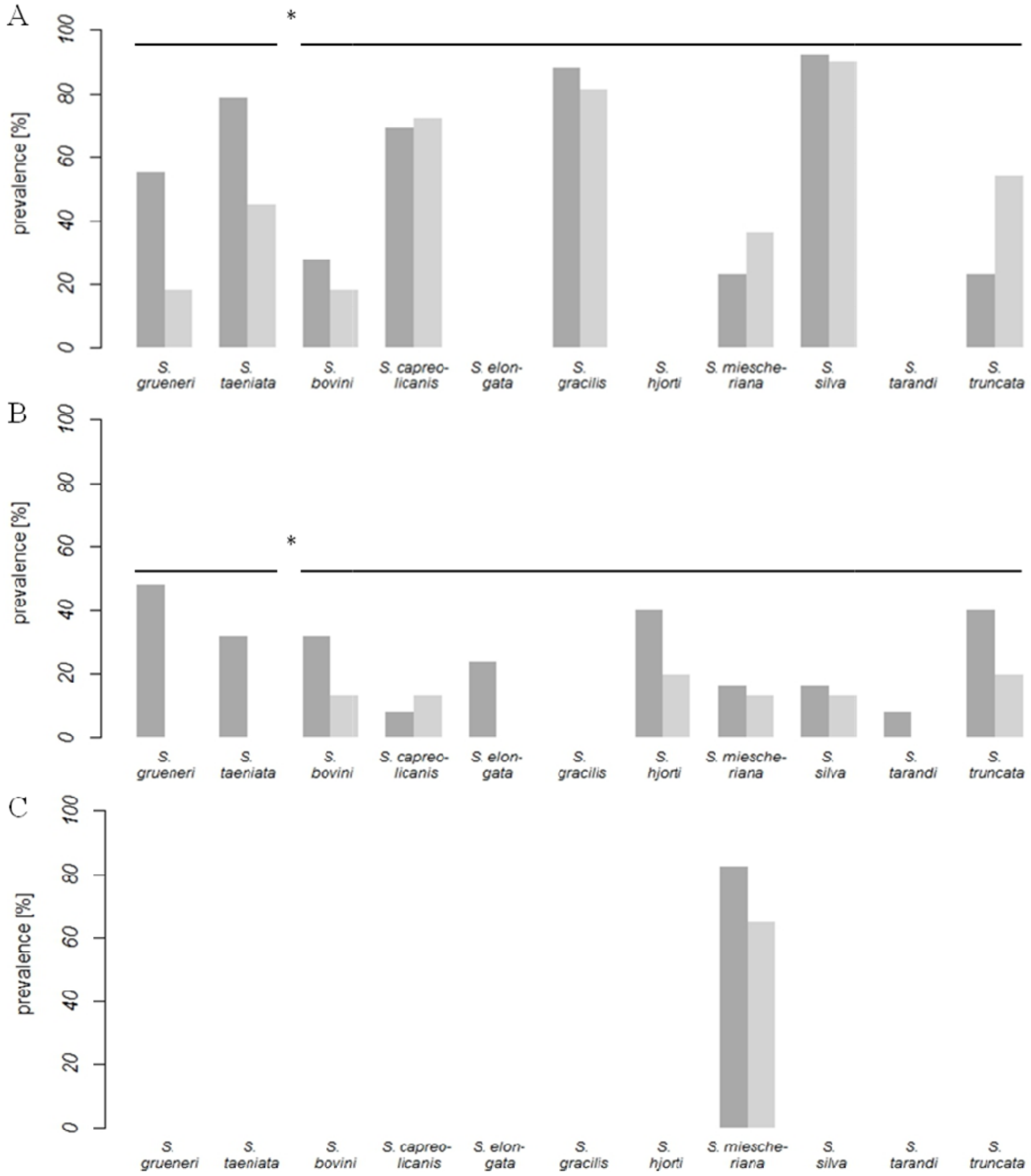


**Suppl. Figure 2:** Normalized prevalences of genetically detected *Sarcocystis* species in ungulates from wolf areas (dark grey) and control areas without wolves (light grey). (A) Roe deer ($n_{WT}$= 21, $n_{CA}$ = 10) were infected with eight distinct *Sarcocystis* species, all of them occurring in both study sites. The prevalence increase of the 'wolf–specialized' species *S. grueneri* and *S. taeniata* was significantly higher than that of all other species (p = 0.043). (B) Red deer ($n_{WT}$ = 10, $n_{CA}$ = 4) were infected with 10 distinct *Sarcocystis* species, four of them exclusively isolated from samples harvested in wolf areas. The prevalence increase of the 'wolf–specialized' species *S. grueneri* and *S. taeniata* was significantly higher than that of all other species (p = 0.044). (C) Wild boar ($n_{WT}$ = 20, $n_{CA}$ = 10) originating from both study sites were infected with a single *Sarcocystis* species.
